# Characteristics of the mechanosensitive bladder afferent activities in relation with microcontractions in male rats with bladder outlet obstruction

**DOI:** 10.1038/s41598-017-07898-y

**Published:** 2017-08-09

**Authors:** Naoki Aizawa, Koji Ichihara, Hiroshi Fukuhara, Tetsuya Fujimura, Karl-Erik Andersson, Yukio Homma, Yasuhiko Igawa

**Affiliations:** 1 Department of Continence Medicine, The University of Tokyo Graduate School of Medicine, Tokyo, Japan; 2 Department of Urology, The University of Tokyo Graduate School of Medicine, Tokyo, Japan; 3 Institute for Regenerative Medicine, Wake Forest University School of Medicine, Winston Salem, NC USA

## Abstract

We investigated the characteristics of bladder mechanosensitive single-unit afferent activities (SAAs) in rats with a bladder outlet obstruction (BOO) and their relationship with bladder microcontractions. Male Wistar rats were divided into Sham and BOO groups. Four or 10 days after the surgery, rats were anesthetized with urethane. The SAAs of Aδ- or C-fibers from the L6 dorsal roots were recorded during bladder filling. The BOO group showed a higher number of microcontractions and lower SAAs of Aδ-fibers compared with those of the Sham group. These findings were significant at day 10 post-operatively. In contrast, SAAs of C-fibers were not significantly different between the groups at either day 4 or 10. In the BOO group at day 10, the SAAs of both Aδ- and C-fibers at the “ascending” phase of microcontractions were significantly higher than those at the other phases (descending or stationary), and a similar tendency was also observed at day 4. Taken together, during bladder filling, the bladder mechanosensitive SAAs of Aδ-fibers were attenuated, but SAAs of both Aδ- and C-fibers were intermittently enhanced by propagation of microcontractions.

## Introduction

Detrusor overactivity (DO) has been defined as “a urodynamic observation characterized by involuntary detrusor contractions during the filling phase which can be spontaneous or provoked”^1^, and is commonly associated with the overactive bladder syndrome (OAB)^2^. Although the similarities are questionable^3^, a microcontraction during the storage phase in rodents has been widely used as a surrogate parameter for DO, and these microcontractions are frequently observed in pathophysiological conditions, especially in animal models of bladder outlet obstruction (BOO)^3, 4^. In isolated whole bladder preparations of a rat model of BOO, coordination of micromotions (similar to the microcontractions) was enhanced by stretch, leading to increased pressure fluctuations^5^. In addition, in women with OAB, micromotions were enhanced and concomitantly observed with urinary urgency during filling cystometry (CMG)^6^. These previous studies suggest that the microcontractions or micromotions may partly contribute to the development of urgency in humans with OAB. This has also been discussed in a recent review^7^.

Bladder afferent nerves are composed by Aδ- and C-fibers. A previous study in cats revealed that more than 90% of C-fibers do not respond to normal bladder distension, being so called “silent” fibers^8^. However, at least in rats, C-fibers can respond to normal bladder distension like Aδ-fibers, although they may also fulfill a potentially different role in the bladder sensory function in response to abnormal stimuli^9–11^. We previously demonstrated that bladder microcontractions may be related to the bladder mechanosensitive Aδ-fiber activities even in normal rats^12^. However, so far, there has been no study directly investigating the possible relationship between the microcontractions and sensory afferent transduction in pathophysiological conditions such as BOO. Thus, in the present study, we investigated the characteristics of bladder mechanosensitive single-unit afferent activities (SAAs) in male rats with BOO, and their relationship with bladder microcontractions.

## Results

### CMG measurements (at day 4)

**Figure 1:**
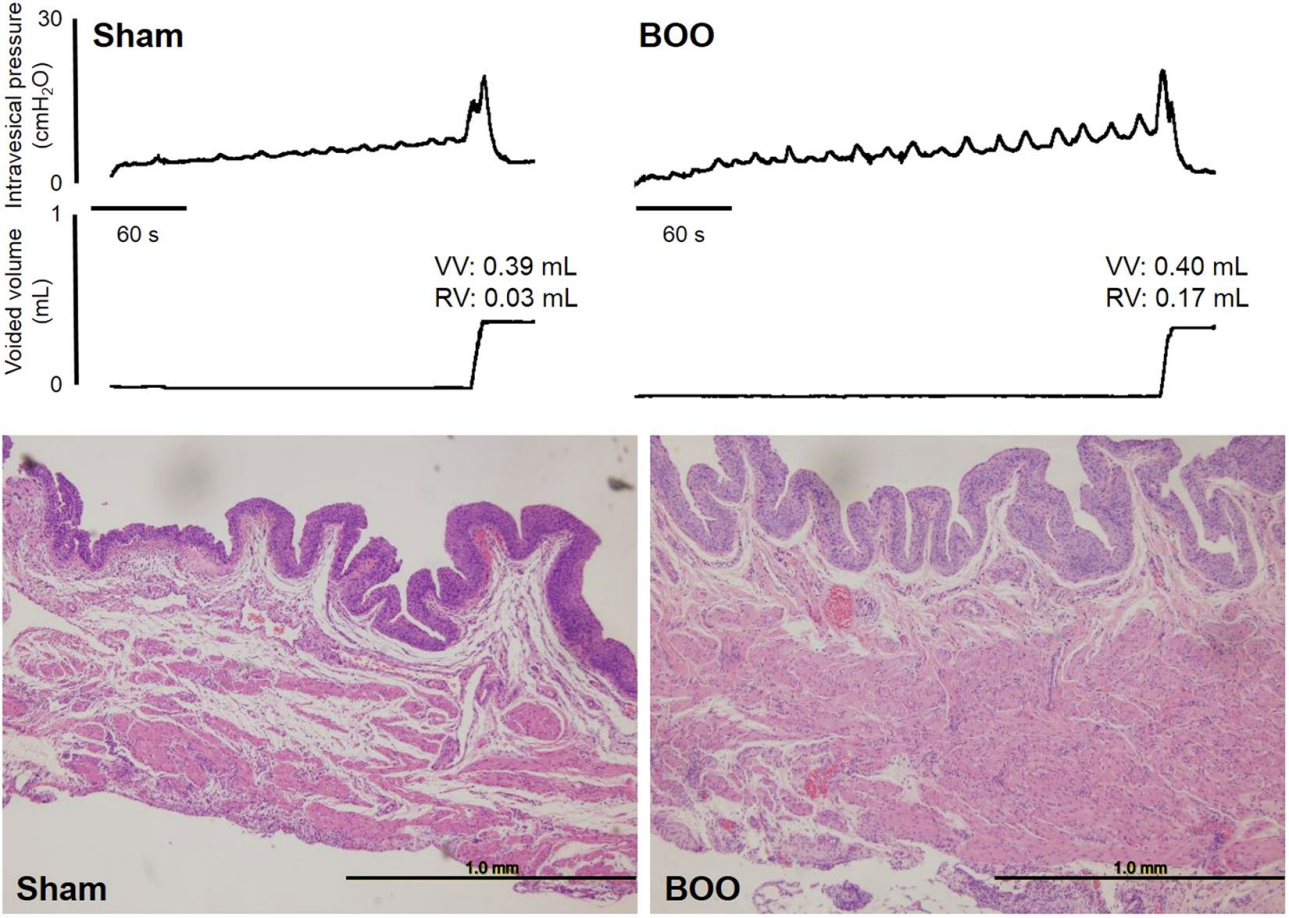
Representative CMG traces (upper traces) and microscopic images with HE staining of the bladder (lower traces) in the Sham and BOO rats at day 4 post-operatively. VV: voided volume, RV: residual volume.

In the present study, we further investigated CMG parameters at day 4, and found that the number and amplitude of NVCs in the BOO rats were significantly higher than in the Sham rats, whereas there were no significant differences between the groups in the other parameters (Fig. 1 upper traces, Table 1). In a previous study, we analyzed CMG parameters at day 10 postoperatively in our BOO model and found that bladder capacity (BC), residual volume (RV), and the mean amplitude and number of non-voiding contractions (NVCs) in the BOO rats were significantly higher than in the Sham rats^13^. However, in this study, CMG parameters were analyzed using only the third measurement in the three-times repeated CMG measurements. When the parameters were reanalyzed by the same methods used in the present study (Supplemental table) we found similar changes in the number and amplitude of NVCs as at day 4.

**Table 1:** CMG parameters in Sham and BOO groups at day 4 post-operatively.

	N	BP (cmH_2_O)	TP (cmH_2_O)	MP (cmH_2_O)	VV (mL)	RV (mL)	BC (mL)	Qave (mL/s)	Number of NVCs (times)	Amplitude of NVCs (cmH_2_O)
Sham	7	2.04 ± 0.36	9.52 ± 0.77	19.16 ± 1.02	0.54 ± 0.08	0.12 ± 0.06	0.66 ± 0.10	0.07 ± 0.01	2.29 ± 0.71	2.93 ± 0.21 (6)
BOO	6	3.39 ± 1.03	11.89 ± 2.00	23.37 ± 4.02	0.70 ± 0.21	0.15 ± 0.07	0.85 ± 0.20	0.08 ± 0.02	6.44 ± 1.45*	3.82 ± 0.25*

The values were expressed as mean ± SEM.

*P < 0.05: significant difference from the Sham rats (unpaired Student’s *t*-test).

The number in a parenthesis indicates the number of Sham rats that were observed the NVCs.

N = number of animals; BP = basal pressure; TP = threshold pressure; MP = maximm pressure during micturition; VV = voided volume; RV = residual volume; BC = bladder capacity; Qave = mean uroflow rate; NVCs = non-voiding contractions.

### Histological findings (at day 4) and bladder weights (at day 4 and 10)

At day 4, the bladders in the BOO rats showed remarkable thickening of the detrusor muscle layer compared with the Sham rats (Fig. 1 lower traces), which was similar to our previous findings observed at day 10^13^. At day 4 and 10, the mean bladder weight in the BOO rats was significantly higher than in the Sham rats (day 4: 160.9 ± 18.1 mg vs. 94.5 ± 5.0 mg, *P* < 0.01; day 10: 190.9 ± 14.5 mg vs. 82.7 ± 2.3 mg, *P* < 0.001).

### SAA measurements (at day 4 and 10)

During the SAAs measurements, bladder compliance in the BOO rats at day 10 was significantly higher than in the Sham rats, whereas no such differences were found at day 4 (Table 2). The number of microcontractions in the BOO rats was significantly higher than in the Sham rats at day 10, whereas there was no significant difference in the number of microcontractions between the Sham and BOO rats at day 4, although a similar tendency was observed (Table 2).

**Table 2:** Characteristics of CVs of Aδ- and C-fibers, bladder compliances, and the number and amplitude of microcontractions in Sham and BOO groups at day 4 and day 10 post-operatively.

Day 4		Sham rats (N = 10)		BOO rats (N = 10)	
		Aδ-fibers	C-fibers	Aδ-fibers	C-fibers
CVs (m/s)		7.69 ± 1.61 (n = 18)	1.91 ± 0.08 (n = 19)	7.40 ± 1.45 (n = 17)	1.91 ± 0.10 (n = 14)
Bladder compliances (mL/cmH_2_O)		0.0138 ± 0.0012		0.0202 ± 0.0046	
Microcontractions	Numbers (times/min)	0.629 ± 0.251		1.496 ± 0.425	
	Amplitude (cmH_2_O)	2.581 ± 0.126 (N = 7)		2.586 ± 0.161	
**Day 10**		**Sham rats** (**N** = **12**)		**BOO rats** (**N** = **12**)	
		**Aδ-fibers**	**C-fibers**	**Aδ-fibers**	**C-fibers**
CVs (m/s)		6.07 ± 1.91 (n = 14)	1.75 ± 0.12 (n = 14)	4.93 ± 0.80 (n = 16)	1.93 ± 0.09 (n = 15)
Bladder compliances (mL/cmH_2_O)		0.0160 ± 0.0014		0.0312 ± 0.0049*	
Microcontractions	Numbers (times/min)	0.095 ± 0.080		1.208 ± 0.179***	
	Amplitude (cmH_2_O)	2.314 ± 0.112 (N = 3)		2.737 ± 0.1678	

The values were expressed as mean ± S.E.M.

*P < 0.05, ***P < 0.001: significant differences from the Sham rats (unpaired Student’s *t*-test). N = number of animals, n = number of nerve fibers.

The number (n) in a parenthesis indicates the number of fibers investigated in this study.

The number (N) in a parenthesis indicates the number of rats that were observed the microcontractions.

**Figure 2:**
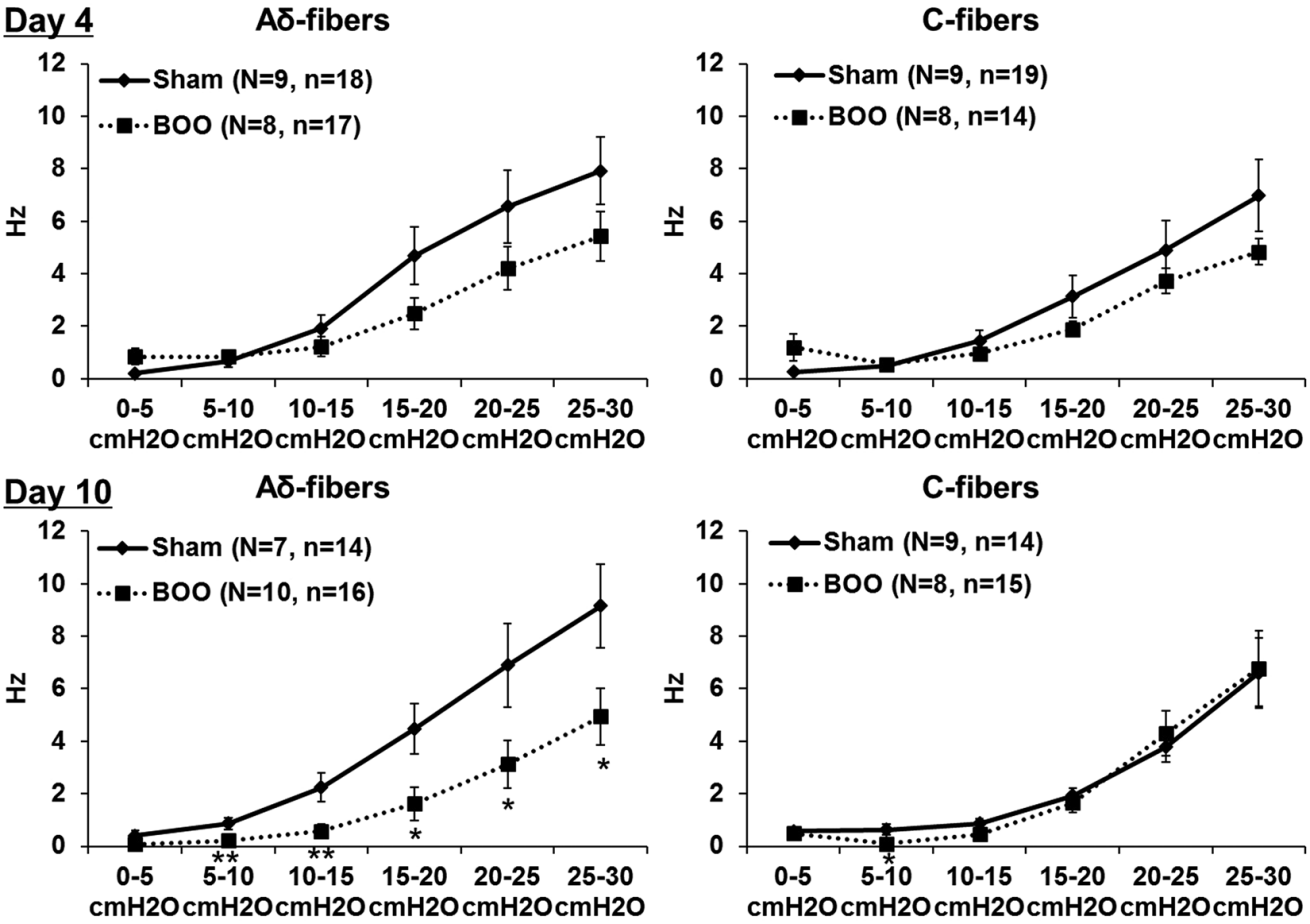
Influence of BOO-induction on mechanosensitive SAAs of the Aδ- and C-fibers. The horizontal and vertical axes indicate the intravesical pressure and firing rate of SAAs, respectively. Values are expressed as mean ± SEM. *P < 0.05, **P < 0.01: significant differences from the Sham rats (unpaired Student’s *t*-test). On both study time-points (day 4 and 10), the BOO rats showed lower SAAs of Aδ-fibers than the Sham rats. These differences were more remarkable and significant at day 10 (Left panels). In contrast, SAAs of C-fibers were not remarkably and significantly different between the Sham and BOO rats (Right panels).

**Figure 3:**
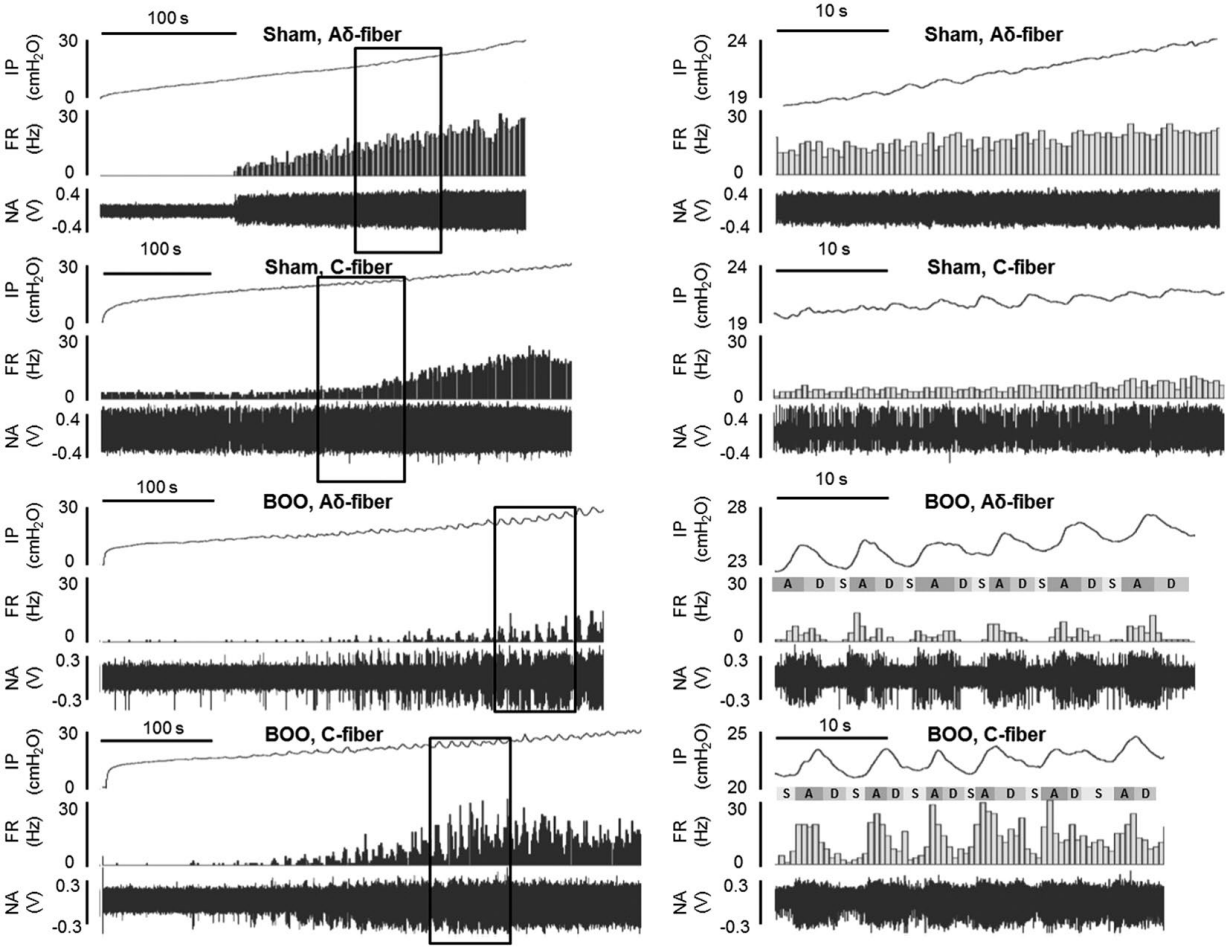
Representative traces of intravesical pressure and the firing rate of the Aδ- and C-fiber in the Sham (upper traces) and BOO rats (lower traces) at day 10. Each square in left panel is corresponding to the right panel. IP: intravesical pressure, FR: firing rate, NA: nerve activity. A: ascending phase, D: descending phase, S: stationary phase. During the measurements, intermittently enhanced SAAs of both Aδ- and C-fiber responses synchronized with bladder microcontractions were frequently observed, especially at the ascending phases, in the BOO rats, but not in the Sham rats.

**Figure 4:**
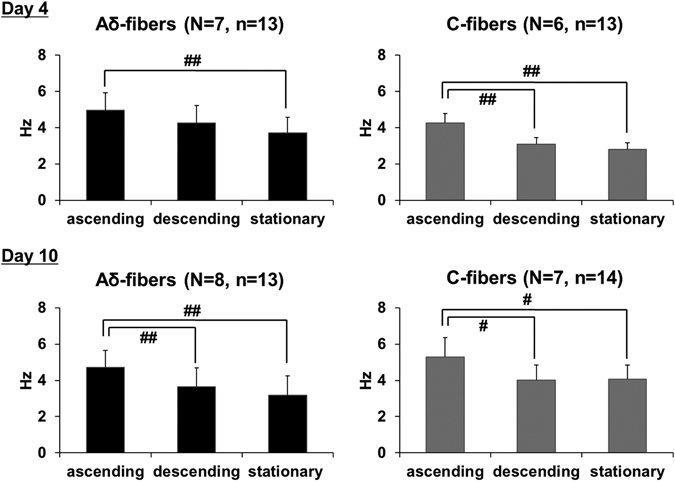
SAAs responses in the BOO rats during three phases of the microcontractions on filling CMG. The vertical axis indicates firing rate of SAAs. Values are expressed as mean ± SEM. ^#^ *P* < 0.05, ^##^ *P* < 0.01: significant differences between each phase (repeated measures ANOVA followed by Tukey’s multiple comparison test).

One hundred and twenty-seven single afferent fibers (more than 14 fibers in each group) were isolated, and there were no significant differences in conduction velocities (CVs) in either Aδ-fibers or C-fibers between the Sham and BOO rats (Table 2). On both study time-points (day 4 and 10), the BOO rats showed lower SAAs of Aδ-fibers than the Sham rats. These differences were more remarkable and significant at day 10, in which lower SAAs were observed throughout the entire filling phase, whereas those at day 4 only appeared during the last half of the filling phase (left panels in Fig. 2). In contrast, SAAs of C-fibers were not remarkably and significantly different between the Sham and BOO rats although slight differences were observed at day 4 (Right panels in Fig. 2). During the measurements, intermittently enhanced SAAs responses synchronized with bladder microcontractions, especially at the ascending phase, were frequently observed in the BOO rats, but not in the Sham rats (Fig. 3). The SAAs of both Aδ- and C-fibers during the ascending phase of microcontractions were significantly higher than those during the other phases (descending and stationary) at day 4 and 10 except those of Aδ-fibers between the ascending and descending phases at day 4 (Figs 3 and 4). Such higher afferent activities during the ascending phase of microcontractions were found in 86% (6/7 rats) and 100% (6/6 rats) for Aδ- and C-fibers, respectively, at day 4, and in 100% (8/8 rats) and 86% (6/7 rats) for Aδ- and C-fibers, respectively, at day 10.

## Discussion

In the SAAs measurements of the present study, no reflex arc through the L6 dorsal roots was preserved, thus the rats could not void. Therefore, we used the term NVCs in CMG and microcontractions in SAAs measurements. It is conceivable that both contractions are similar, and they have been demonstrated to be of myogenic origin^4^. The nature and importance of myogenic microcontractions during the bladder filling phase have been discussed by many investigators. Microcontractions were originally proposed as a motor component of a motor/sensory system^14–17^, and their importance for urinary storage and voiding dysfunction such as overactive bladder and detrusor underactivity was recently discussed^7^. Microcontractions have been found to be enhanced in pathological situations such as BOO^18^.

The CMG measurements in the present study demonstrated that the BOO rats showed increases in the number and amplitude of NVCs at day 4 post-operatively. Our previous^13^ and re-analyzed data (Supplemental table) by using the same CMG measurements showed that the number and amplitude of NVCs in the BOO rats were increased also at day 10. In addition, in the present SAAs measurements, the BOO rats showed an increase in the number of microcontractions, and these contractions were conspicuous at day 10 rather than day 4. These findings may partly support a previous study, in which increased coordination of micromotion waves with stretch and the consequent enhancement of intravesical pressure fluctuations following BOO may be involved in the pathophysiology of DO^5^. Moreover, the BOO rats showed higher bladder weights compared with the sham rats accompanied with thickening of the detrusor muscle layer at day 4, and such changes were more pronounced at day 10^13^.

In the pathophysiological condition of lower urinary tract dysfunction, bladder efferent function can be deteriorated, e.g. due to peripheral denervation^19–23^. In this condition, loss of efferent function may enable unregulated microcontraction activity, and afferent stimulation, predisposing to urinary urgency. During the storage phase, the bladder is not totally quiescent. In humans, there are low amplitude microcontractions in the bladder, which have barely-detectable effect on intravesical pressure^6^. In addition, Parsons *et al*. demonstrated that microcontractions (termed micromotions in the article) occurred over the bladder surface, with variable correlation with intravesical pressure fluctuations (microcontractions) in the pig bladder^24^. Exaggerated microcontractions are seen in animal models of lower urinary tract pathologies. For example, Drake *et al*. demonstrated that an increased coordination of microcontractions in isolated bladder strips with stretch and the consequent enhancement of bladder microcontractions in the isolated rat bladder after creating BOO^5^. These previous reports suggest that microcontraction propagation and general bladder tone at any given moment are key factors determining whether autonomous bladder activity results in proportionate intravesical pressure fluctuations.

The present BOO rats showed lower SAAs of Aδ-fibers and this finding was more conspicuous at day 10 than day 4, whereas C-fibers in the BOO rats did not show such lower activities compared with the Sham rats at either day 4 or day 10. These results suggest that BOO causes time-dependent denervation in myelinated Aδ-fibers, but not in unmyelinated C-fibers, of the bladder mechanosensitive afferent nerves at least within 10 days after mechanically creating BOO. Saito *et al*. demonstrated a linear correlation between the severity of obstruction and the degree of reduction in blood flow to the bladder^25, 26^, these changes leading to hypoxia and/or ischemia. In ischemic rabbit bladder tissue with atherosclerosis, Azadzoi *et al*. showed degenerating and collapsed axons and Schwann cells surrounded by dense connective tissue and splitting of the myelin sheaths^27^. In addition, Dahlin *et al*. reported that the thinner myelinated fibers were more susceptible to deprivation of oxygen under ischemic condition than the thicker ones, whereas unmyelinated fibers were resistant to ischemic induction^28^. These studies support our findings that mechanosensitive myelinated Aδ-fiber activity was attenuated following BOO condition.

We further investigated the characteristics of the SAAs related with microcontractions, and showed the SAAs of both Aδ- and C-fibers under the BOO condition were enhanced during the ascending phases. Even in un-treated (normal) rats, microcontractions and their synchronized SAAs have been observed in Aδ-fibers, but not in C-fibers. However, these microcontractions were rarely observed and relatively small^12^. In the present study with the BOO rats, more sustained and exaggerated SAAs of both Aδ- and C-fibers synchronized with microcontractions were demonstrated, suggesting that the myogenic bladder microcontractions promote the mechanosensitive bladder afferent activities at least under the BOO condition. Aδ-fibers are mainly located in the smooth muscle layer, whereas C-fibers distribute throughout the bladder wall from the smooth muscle layer to the urothelium^29^. It has been demonstrated that microcontractions provoked by BOO were of myogenic origin^4^, which may be strongly related with the function of the smooth muscle layer. Thus, it is reasonably assumed that propagation of microcontractions under the mechanical BOO condition causes intermittently enhanced SAAs of Aδ-fibers. Interestingly, similar SAAs enhancement by propagation of microcontractions was observed also in C-fibers in the present study. C-fibers in the bladder appear to be mechano-insensitive and “silent” (i.e., responding only to noxious and inflammatory stimuli), and may not participate in normal micturition in cats^8, 30^. In contrast, previous reports indicated that in rats, some C-fibers may be volume receptors and may not respond to bladder contractions^31^. Although we need further investigations, C-fibers may potentially fulfill a key role in the bladder abnormal sensation in response to microcontractions under pathophysiological conditions, i.e. it may be speculated that such intermittently enhanced afferent activities synchronized with microcontractions can link to the abnormal bladder sensation such as urgency.

In the present study, there is a limitation of the way of fiber classification, which was solely based on the CV value, i.e. Aδ- and C-fibers. Although this simple classification is helpful for our understanding of the afferent outflow from the bladder, there are several histologically different fiber types, regional variations in innervation and alterations of fiber properties following pathological changes. Thus, the present findings need to be considered in the light of all the new ideas that are emerging in relation to the structural and functional complexities of the bladder.

It has been suggested that myogenic microcontractions during the storage phase contribute to the development of urgency^6, 32^. To our knowledge, the present study is the first direct demonstration of a relationship between the mechanosensitive afferent fiber activities and bladder microcontractions under pathophysiological conditions. Although there are limitations regarding the experimental setup such as under urethane-anesthesia^3^, and acute severe BOO compared to chronic slowly progressive BOO from benign prostatic enlargement in human, if valid in humans, this might be supports the view that microcontractions contribute to the development of urgency and/or DO associated with BOO^6^.

In conclusion, there is a relation between the mechanosensitive afferent fiber activities and bladder microcontractions in a pathophysiological condition such as BOO. During bladder filling, the bladder mechanosensitive SAAs of Aδ-fibers were attenuated, but SAAs of both Aδ- and C-fibers were intermittently enhanced by propagation of microcontractions.

## Methods

### Ethical approval and animals

The protocol was approved by the Institutional Animal Care and Use Committees of the University of Tokyo and was in line with the NIH guidelines for the care and use of experimental animals. Fifty-seven adult male Wistar rats were used (11 weeks old, 254–295 g, Japan SLC, Shizuoka, Japan). The rats were maintained under standard laboratory conditions with a 12:12 h light: dark cycle, and free access to food and water. Maintenance and killing of the animals followed principles of good laboratory practice in compliance with national laws and regulations. The animals were killed humanely by overdose of anesthesia.

### Surgical procedures for creating BOO and experimental schedule

Surgery to create partial BOO was done as described in previous reports^13, 33, 34^. In brief, the proximal urethra was tightly ligated with a steel rod (1.2 mm in diameter), then the rod was removed. This ligation was kept to the end of the experiments. Sham rats received similar surgery without the urethral ligation. Each animal was housed in a separate cage. CMG measurements and histological examinations were performed at day 4 post-operatively (Sham: N = 7, BOO: N = 6), when the BOO rats showed an increase in voiding frequency and decreases in mean uroflow rate (Qave) and voided volume per micturition (VV) based on our previous findings with voiding behavior measurements in a metabolic cage^13^. In separate animals, SAAs measurements were performed at day 4 (N = 10, in each group) and 10 (N = 12, in each group).

### CMG measurements and histological examinations

The methods for CMG measurements were slightly modified from our previous study^13^. In brief, at the same day of the surgery for creation of Sham or BOO, a PE-50 catheter (Clay Adams, Parsippany, NJ) with a cuff was implanted into the bladder. Four days after the catheter-implantation (on day 4), CMG measurements in a conscious restrained condition were performed. Saline was instilled into the bladder at a rate of 6 mL/h until micturition occurred, and voided urine was collected in a cup placed just under the penis and weight of voided urine was measured. After each micturition, the bladder catheter was disconnected and the post-void residual was collected by natural dropping through the catheter for 10 min and measured. CMG recordings were repeated 3 times and the following parameters were averaged and analyzed: basal pressure (BP: minimum intravesical pressure), threshold pressure (TP: intravesical pressure at the onset of micturition), maximum pressure (MP: maximum intravesical pressure during micturition), VV, RV (the volume of urine collected after micturition), BC (VV + RV), Qave (VV/voiding time), and mean amplitude and the number of NVCs. NVCs were defined as bladder contractions without micturition, the amplitudes of which were more than 2 cmH_2_O, observed for 3 min before micturition.

In some animals (four Sham and five BOO rats) of the CMG measurements, the whole bladder was isolated at the end of the experiments, subsequently fixed in 4% paraformaldehyde-PBS, then embedded in paraffin and cut into 3 µm sections. Finally, the bladder specimens were evaluated with HE staining.

### SAAs measurements

SAAs measurements were performed as described previously under urethane anesthesia (1.2 g/kg, intraperitoneally)^35^. In brief, after a pair of silver stimulation electrodes was placed around the left pelvic nerve, both L6 dorsal roots were cut near their entrance into the spinal cord. Unitary action potentials of mechanosensitive bladder afferent nerve fibers isolated from left L6 dorsal roots were identified as SAAs by electrical stimulation of the pelvic nerve and by bladder distension with saline via an inserted bladder catheter (PE-50). The SAAs were grouped on the basis of their CV, i.e. those with a CV of less than 2.5 m/s were considered to correspond to unmyelinated C-fibers, whereas those with a CV of 2.5 m/s or greater were considered to correspond to myelinated Aδ-fibers. SAAs and intravesical pressure were recorded during constant saline-instillation into the bladder. Based on the values of BC obtained from the CMG measurements in our previous^13^ and the present studies, saline-instillation rate was applied at 6 ml/h in the Sham (at day 4 and 10) and BOO rats (at day 4), and at 10 ml/h in the BOO rats (at day 10). Such different rates of saline-instillation may affect the development of microcontractions or SAAs, but our preliminary investigation showed that there were no remarkable differences in the microcontractions or SAAs between the different rates (data not shown). The filling continued until an intravesical pressure of 30 cmH_2_O was achieved, and the bladder compliance was simply calculated between the start and end of the saline-instillation into the bladder during measurements. The SAAs were expressed as the firing rates (Hz) and evaluated in relation to intravesical pressure at each 5 cmH_2_O-interval.

**Figure 5:**
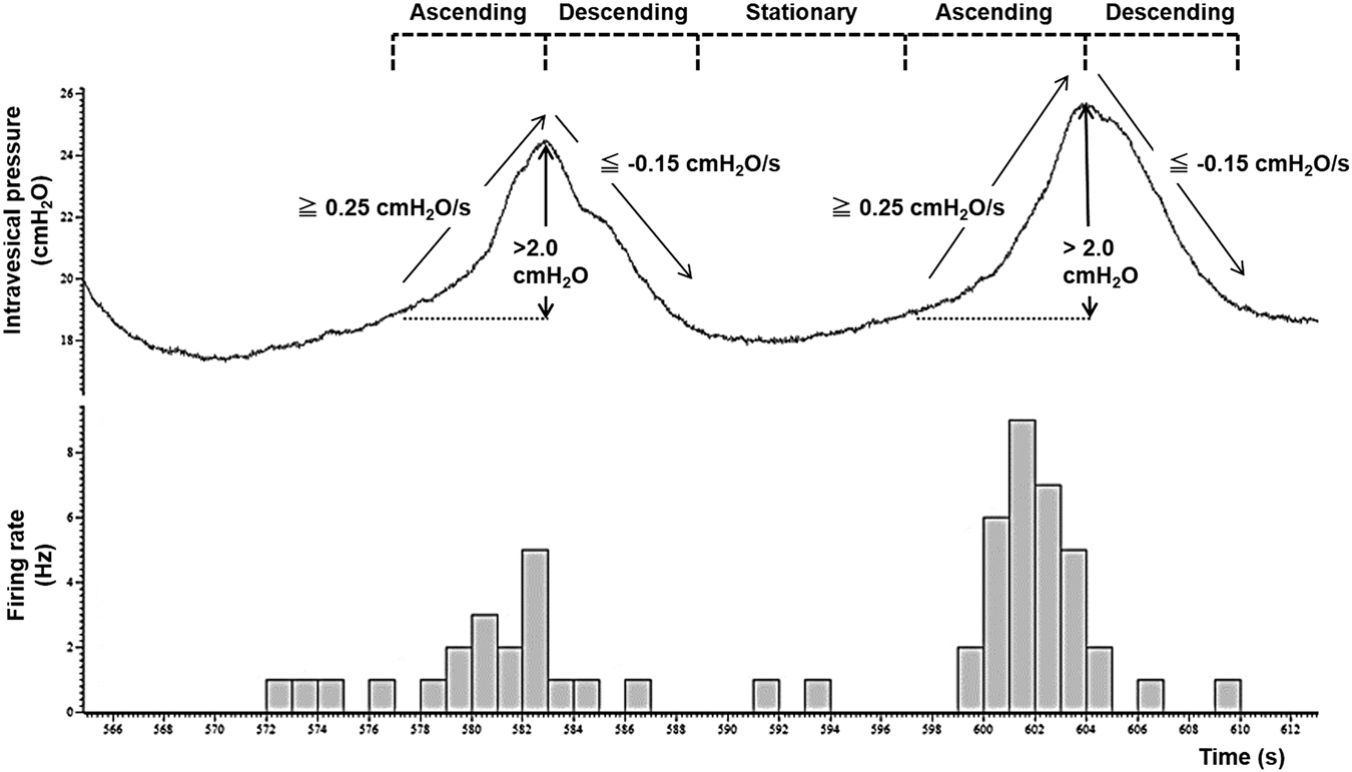
Definition of the microcontractions (ascending and descending phases) and stationary phase in the present study. Upper trace is intravesical pressure, and lower trace is firing rate of SAAs. The microcontractions were defined as the contractions having more than 2 cmH_2_O of amplitude, and pressure was ascending at 0.25 cmH_2_O/s or more, and then descending at 0.15 cmH_2_O/s or more. And the portions between these phases were defined as the stationary phases.

A microcontraction was defined as a contraction with an amplitude of more than 2 cmH_2_O, and where intravesical pressure was ascending at 0.25 cmH_2_O/s or more and then descending at 0.15 cmH_2_O/s or more. The microcontraction was divided into two phases: “ascending” and “descending”. The portion between the two microcontractions was termed as the “stationary” phase (Fig. 5).

### Statistical Analyses

All data are expressed as mean ± SEM. The results between the Sham and BOO rats were analyzed using an unpaired Student’s *t*-test. To evaluate the SAAs related with microcontractions, repeated measures ANOVA followed by Tukey’s multiple comparison test was used. *P* values < 0.05 were considered statistically significant.

## Electronic supplementary material

Supplementary information
