# Supplementary material for: Characteristics of the mechanosensitive bladder afferent activities in relation with microcontractions in male rats with bladder outlet obstruction
Source: Sci Rep. 2017 Aug 9;7:7646. doi: 10.1038/s41598-017-07898-y (PMC5550413; doi:10.1038/s41598-017-07898-y)
Supplement: Supplementary file 1 — Supplementary information [file 41598_2017_7898_MOESM1_ESM.pdf]

Supplemental Table

Manuscript title:

Characteristics of the mechanosensitive bladder afferent activities in relation with microcontractions in male rats with bladder outlet obstruction

Authors and institutions:

Naoki Aizawa[1*], Koji Ichihara[1], Hiroshi Fukuhara[2], Tetsuya Fujimura[2], Karl-Erik Andersson[3], Yukio Homma[2], Yasuhiko Igawa[1]

1. *Department of Continence Medicine, The University of Tokyo Graduate School of Medicine, Tokyo, Japan*
2. *Department of Urology, The University of Tokyo Graduate School of Medicine, Tokyo, Japan*
3. *Institute for Regenerative Medicine, Wake Forest University School of Medicine, Winston Salem, NC, USA*

*Corresponding author:
Naoki Aizawa
Department of Continence Medicine, The University of Tokyo Graduate School of Medicine, 7-3-1, Hongo, Bunkyo-ku, Tokyo, 113-8655, Japan
Tel & Fax: +81-3-5800-9792
E-mail: naoki-aizawa@hotmail.co.jp

CMG parameters in Sham and BOO groups at day 10 post-operatively, re-analysis of reference 13 (Sugiyama et al., 2015)

	N	BP (cmH_2O)	TP (cmH_2O)	MP (cmH_2O)	VV (mL)	RV (mL)	BC (mL)	Qave (mL/s)	Number of NVCs (times)	Amplitude of NVCs (cmH_2O)
**Sham**	22	5.02 ± 0.44	16.29 ± 1.36	37.31 ± 1.81	0.78 ± 0.06	0.12 ± 0.03	0.90 ± 0.08	0.14 ± 0.01	1.97 ± 0.32	3.58 ± 0.19 (21)
**BOO**	22	4.19 ± 0.30	11.48 ± 1.13**	39.79 ± 2.85	0.73 ± 0.08	1.61 ± 0.28**	2.35 ± 0.26**	0.05 ± 0.01**	5.53 ± 0.35**	5.81 ± 0.45**

Three CMG recordings were averaged and parameters were analyzed.

The values were expressed as mean ± SEM

***P*<0.01: significant differences from the Sham rats (unpaired Student's *t*-test).

The number in a parenthesis indicates the number of Sham rats that were observed the NVCs.

N = number of animals; BP = basal pressure; TP = threshold pressure; MP = maximm pressure during micturition; VV = voided volume; RV = residual volume; BC = bladder capacity; Qave = mean uroflow rate; NVCs = non-voiding contractions
